# Spatiotemporal analysis of regional TIA trends

**DOI:** 10.3389/fneur.2022.983512

**Published:** 2022-08-22

**Authors:** Andrew Kawai, Samuel Hui, Richard Beare, Velandai K. Srikanth, Vijaya Sundararajan, Henry Ma, Thanh G. Phan

**Affiliations:** ^1^School of Clinical Sciences at Monash Health, Stroke and Aging Research Group, Clinical Trials, Imaging & Informatics Division, Melbourne, VIC, Australia; ^2^Department of Neurology, Monash Health, Melbourne, VIC, Australia; ^3^Department of Medicine, Monash Health, Melbourne, VIC, Australia; ^4^Academic Unit, Peninsula Clinical School, Central Clinical School, Monash University, Melbourne, VIC, Australia; ^5^Department of Geriatric Medicine, Peninsula Health, Melbourne, VIC, Australia; ^6^Developmental Imaging, Murdoch Children's Research Institute, Melbourne, VIC, Australia; ^7^Department of Public Health, La Trobe University, Melbourne, VIC, Australia; ^8^Department of Medicine, St Vincent's Hospital Melbourne, Melbourne, VIC, Australia

**Keywords:** TIA, spatial regression, spatiotemporal regression, disease mapping, areal unit

## Abstract

**Background:**

There has been a decline in the stroke incidence across high income countries but such knowledge exists at Country or State rather than areal unit level such local government area (LGA). In this disease mapping study, we evaluate if there are local hot spots or temporal trends in TIA rate. Such knowledge will be of help in planning healthcare service delivery across regions.

**Methods:**

Linked hospital discharge data (Victorian Admitted Episodes Dataset or VAED) was used to collect TIA (defined by ICD-10-AM codes G450-G459) cases from 2001 to 2011. The State of Victoria is the second most populous state in Australia, with a population of 6.7 million and can be divided into 79 administrative units or LGA. The data is anonymized and contains residence of the patient in terms of LGA but not exact location. The date of the TIA event when the patient is admitted to hospital is provided in the dataset. The number of TIAs per year was aggregated for each LGA. Standardized TIA ratios were calculated by dividing actual over expected cases for each LGA per year. We used Integrated Nested Laplace Approximation (INLA) to perform spatial and spatiotemporal regression, adjusting for hypertension, sex and population, age (≥60), and socio-economic status (SES) decile within the LGA. The final model was chosen based on the lowest the Deviance Information Criterion (DIC) and Watanabe-Akaike information criteria (WAIC).

**Results:**

Choropleth maps showed a higher standardized TIA ratios in North-West rural region. Compared to the baseline model (DIC 13,159, WAIC 13,261), adding in a spatial random effect significantly improved the model (DIC 6,463, WAIC 6,667). However, adding a temporal component did not lead to a significant improvement (DIC 6,483, WAIC 6,707).

**Conclusion:**

Our finding suggests a statically significant spatial component to TIA rate over regional areas but no temporal changes or yearly trends. We propose that such exploratory method should be followed by evaluation of reasons for regional variations and which in turn can identify opportunities in primary prevention of stroke, and stroke care.

## Introduction

In 2016, the global lifetime risk of stroke from the age of 25 was 24.9%, an increase from 22.8% in 1990, and was almost twice as high for ischaemic than haemorrhagic stroke (1). Stroke incidence, hospitalization rates and mortality for stroke has also been continuing to decline in high-income countries (2, 3). Particularly in the UK and Australia, stroke incidence and survival after stroke has improved over the 1990s/2000s decade (4, 5). At Country or State level, studies of trends in recurrence such as by our group (6) and others (7, 8) document a small decrement in trend in risk of recurrence in early 2000s and more recently plateauing of recurrence rate in the last 5 years (7). In geographically large country such as Australia with great distance between rural and urban areas, it is not known if the observed decrease in total stroke incidence/hospitalization rates and mortality for stroke over time also occurs at a regional level (6).

To plan and ensure fair health care access for all, we need to evaluate if the TIA incidence is different at a regional level (hot spots) and if these geographical patterns change over time and if prevention program needs to be designed for the region given that region's attributes (9). Papers and maps focussing on TIA and stroke prevalence and state of stroke services across the globe are available but focussed works on regional data in Australia for TIA is not available (10–12). This knowledge can help planning of primary and secondary prevention therapy. Investigators have used spatial model to show that the racial differences in cardiovascular health across United States of America (USA) exist predominantly in the Stroke Belt (South Eastern USA) and Stroke Buckle (Northeast region surrounding the Great Lakes) (13). The Stroke Belt region has been subject of many publications over the year trying to explore the cause of this racial disparity in health outcome (14). At a global level, the term global stroke belt has been used to describe the higher prevalence of stroke from Eastern Europe to Russia and China (15). To our knowledge there are no previous attempts with an ecological model to map regional variation in TIA rate in Australia. This issue is understandable given that the approach is methodologically complex and requires significant coding in programming language rather than through commercial statistical program with graphical user interface. This is necessary as there is a needs to account for spatial and temporal correlation in the data (16). For example, spatial correlation can occur among neighboring administrative units (16), termed Local Government Areas (LGA) in Australia. In this study, we perform disease mapping to explore regional trend in TIA hot spots using firstly spatial regression follow by spatiotemporal analysis to better characterize the location of TIA hot spots and changes of these TIA hot spots over time.

## Design and methods

### Setting

The State of Victoria is the second most populous state in Australia, with a population of 6.7 million based on 2016 Census and can be divided into 79 administrative units or LGA (17, 18). Melbourne is the capital city of the State of Victoria and has a population of ~4 million. For this study, the entire State will be separately analyzed. A sensitivity analysis will be performed in a subset of the data exploring if the changes are consistent when the analysis is restricted to Greater Melbourne. In this paper, the term areal unit refers to the fact that data for each LGA is contained within a bounded region.

### Data sources

The retrieved hospital data (Victorian Admitted Episodes Dataset or VAED) has been described previously in a publication on overall trend in TIA rate for the State of Victoria (6). In short, all TIA episodes from linked hospital discharge and Emergency Department (ED) data were extracted for the period between July 1, 2001 to June 30, 2011 based on 4-digit ICD-10 codes for TIA encompassing G450-G459 (19). Only the incident episodes of TIA were collected, ensuring that the case did not present previously with a TIA or stroke (ICD-10-AM codes 1630–1639) in the previous 2 years. The data from VAED is anonymized and contains residence of the patient in terms of LGA but not exact location. As such analysis is performed at areal unit level rather than at point pattern level. The date of the TIA event when the patient is admitted to hospital is provided in the dataset. The number of TIAs per year in each LGA was then calculated. The geocodes of the boundaries of each LGA were retrieved from the Australian Bureau of Statistics for the spatial and spatiotemporal analyses (20).

### Statistical analysis

Standardized TIA ratios were calculated by dividing the actual number of TIA cases for each LGA for each year over the expected number of cases (16). The expected cases for each LGA can be obtained by multiplying raw TIA rate by the population in each LGA. This is the first step toward performing spatial regression. Choropleth (thematic) maps were created to depict standardized TIA ratios over time with the color representing standardized TIA ratio. In this paper, we used red color to highlight higher standardized ratio. Analysis was completed for both all LGAs in the entire State, and LGAs only in Greater Melbourne. Following the creation of raw maps of standardized TIA ratios, spatial regression was performed adjusting for aggregate count of hypertension, sex and population count, proportion of population above 60 and SES decile according to the Index of Relative Socioeconomic Advantage and Disadvantaged (IRSAD) within each LGA.

**Figure 1:**
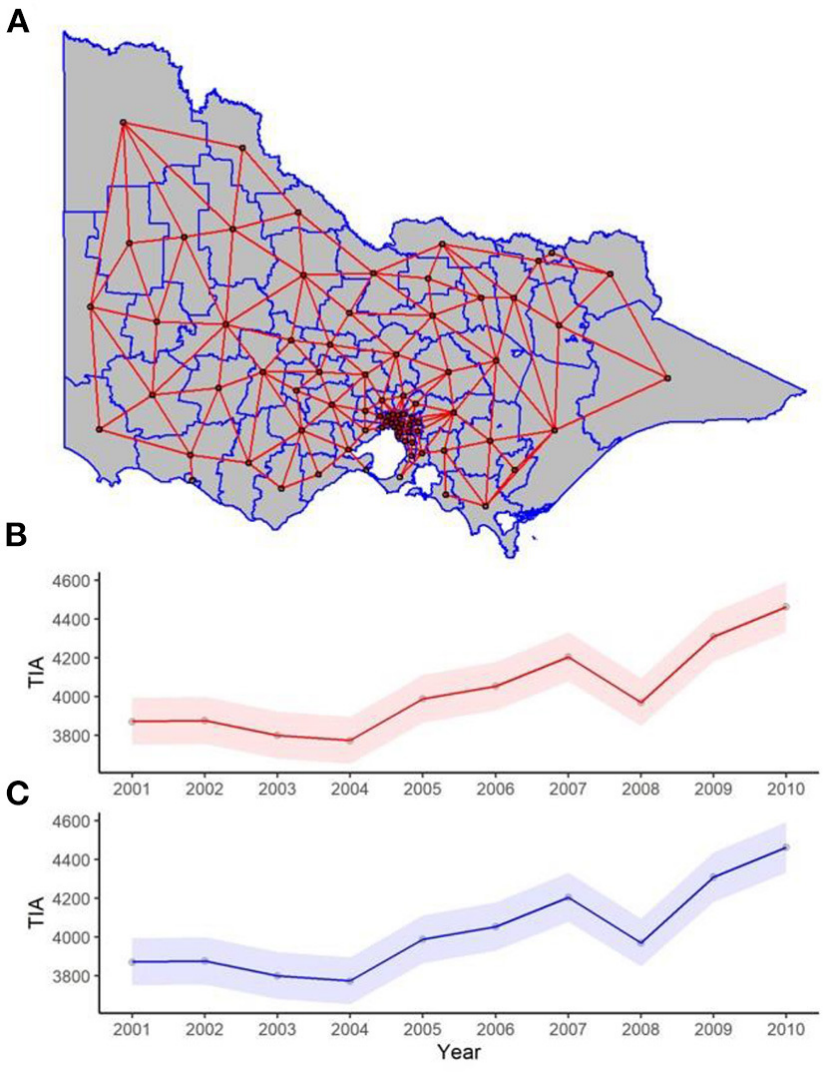
**(A)** Spatial and **(B,C)** temporal weights for spatial and spatiotemporal regression. **(A)** Each red line in the map of the state represents two neighboring LGAs (where two regions share one point in common boundary), and this was used for the spatial weight component of the spatiotemporal regression. The red and blue trend lines of TIAs per year are the posterior distribution of the temporal weights of the **(B)** spatial lag, and **(C)** Leroux models respectively used in the spatiotemporal regression, with 95% confidence interval bands.

The idea in spatial regression is that neighboring regions are similar but distant regions are less so (16). In other words, adjacent regions are not independent but correlated. The nature of this correlation can be defined according to the spatial weight for LGA. The concept of neighbor is defined as any two regions that share a common border. In Figure 1, it can be seen that the centroid of one LGA is connected, regardless of direction, to adjacent centroid. Next, the spatial weight is incorporated into regression analyses analogous to weighted regression. These analyses were performed using a latent Gaussian model known as Integrated Nested Laplace Approximation (INLA) (21). The INLA method provides a fast and efficient approach to approximate the Bayesian posterior distribution compared to prior approaches using other software such as Bayesian inference Using Gibbs Sampling running on Windows operating system (WINBUGS) (21).

A multivariate Poisson model adjusting for hypertension, sex and population count was used as the baseline model. Multiple different spatial regression algorithms were then used to explore the trends in TIA over different LGA (16, 22).

#### Spatial models

1- Fixed = baseline mmodel adjusted to hypertension, sex and LGA population count.
2- Spatial 1 = Intrinsic conditional autoregression/ICAR (or Besag) model. This method uses spatial weight from the neighborhood adjacency matrix (structured random effect) (16). This model may not function well in cases where the areal unit contains small count (of the number of TIA) because of the possibility of over smoothing these smaller areal units.
3- Spatial 2 = Besag-York-Mollie model (BYM). This model extends the intrinsic conditional autoregressive component from above to include an ordinary (unstructured) random effects component for non-spatial heterogeneity (16).
4- Spatial 3 = Leroux et al. model. The Leroux model can be considered as a generalization of ICAR and BYM models as it combines featured of the structured and unstructured random effect component into one (16). This approach attempts to find a balance between the ICAR and BYM models in terms of the local weight for smoothing.
5- Spatial 4 = Spatial lag model. This model is appropriate when the outcome and explanatory data exhibit spatial clustering (22). In this case the outcome is predicted by an additional term in the regression model describing the neighboring region's outcome.

#### Spatiotemporal models

**Figure 2:**
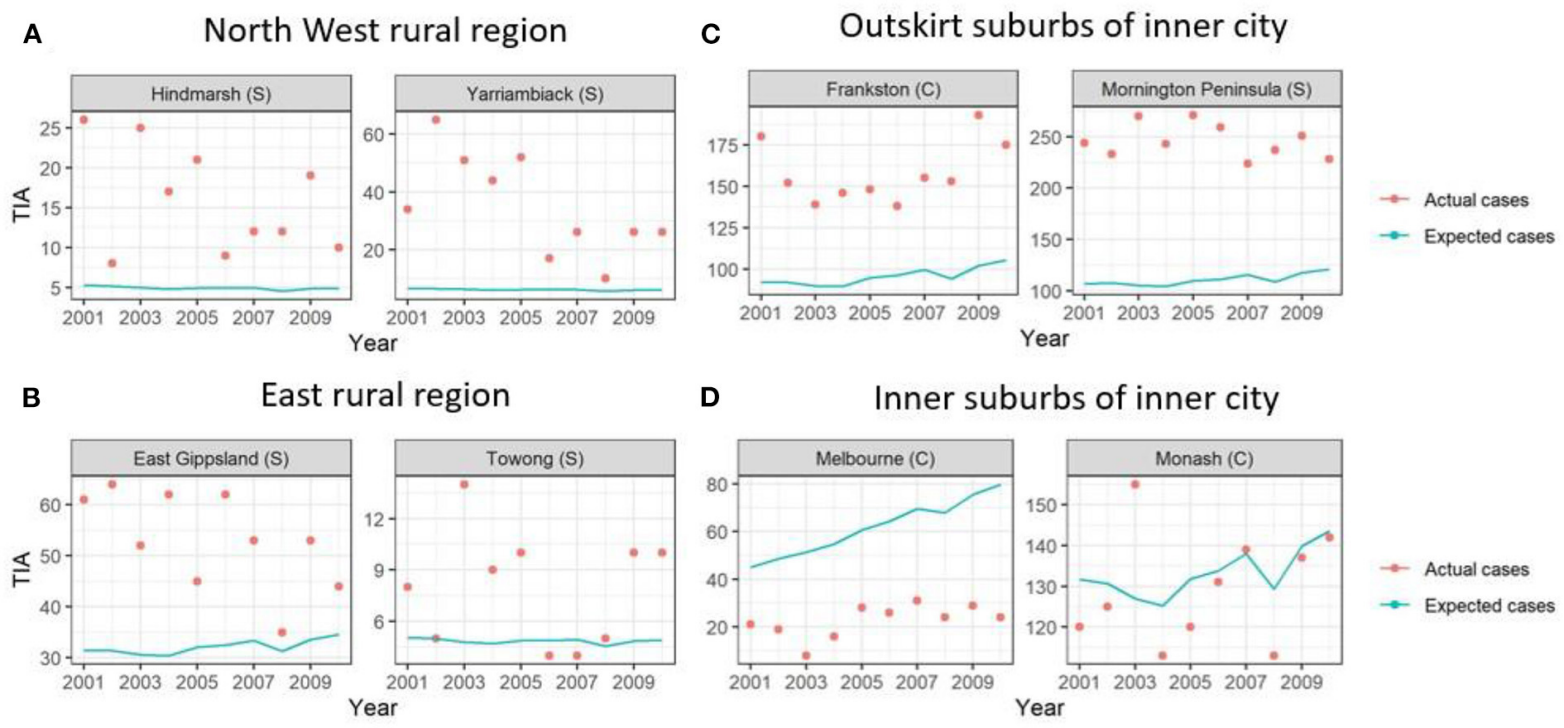
Graphs of example LGAs highlighting actual cases compared to expected cases over 2001-2010. Actual cases (red dots) were higher than the expected cases (blue line) in **(A)** North West rural region and **(B)** East rural region and **(C)** outskirt suburbs of inner city, highlighting TIA hot spots. This is in comparison to the **(D)** inner suburbs of the inner city, where the actual cases were similar to expected cases or lower.

**Figure 3:**
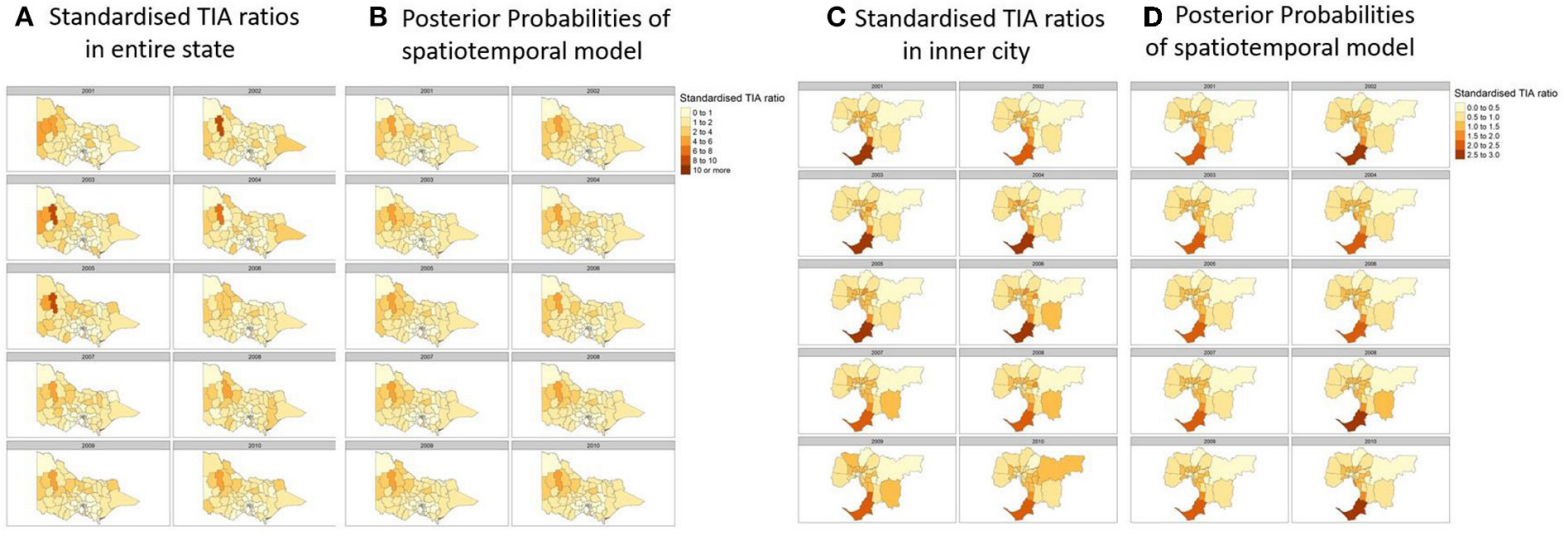
**(A)** Actual Standardized TIA Ratios measured each year in the state. **(B)** Posterior probabilites of the best performing spatiotemporal model utilizing a spatial lag component and temporal component of random walk order of one. **(C)** Actual Standardized TIA Ratios measured each year in the inner cities of the state. **(D)** Posterior probabilites of the best performing spatiotemporal model utilizing a spatial lag component and temporal component of random walk order of one. Covariates used in the regression were hypertension, sex, and population count, proportion of population above 60 and SES decile according to the Index of Relative Socioeconomic Advantage and Disadvantaged (IRSAD).

The spatial model was expanded for the spatiotemporal case by adding a linear component to map for time and time-space interactions (Figures 2, 3) (23, 24). The spatiotemporal models evaluated included:

1- Temporal 1 = Temporal component with random walk of order one. This model applies a temporal term which takes on a random walk through the neighborhood structure (23).
2- Temporal 2 = Temporal component with autoregression of order one. This model include a time lagged value from the previous year (24).

Models were calibrated using Marginal likelihood, Deviance Information Criterion (DIC) and Watanabe-Akaike information criteria (WAIC) (25, 26). The optimal model is one which has the lowest DIC and WAIC. The temporal weights can be seen in Figure 1. All analysis was completed in R programming language (version 4.0.3).

### Sensitivity analysis

**Figure 4:**
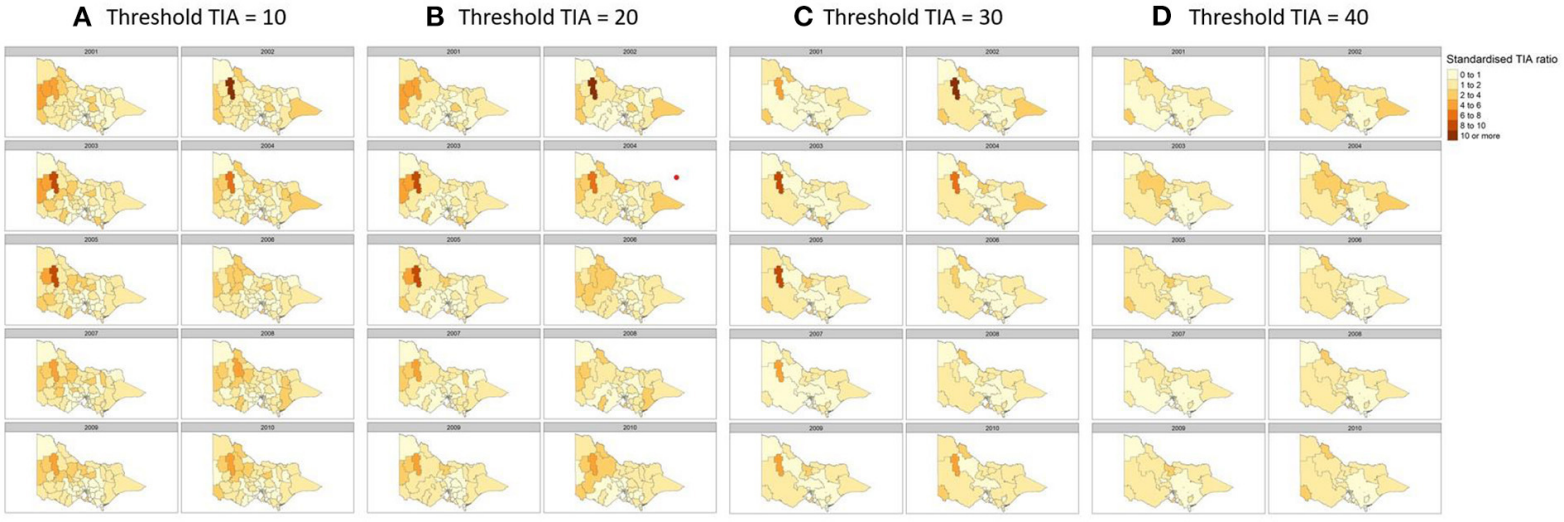
Sensitivity analysis to detect changes in hot spots by merging regions with TIA count of **(A)** 10, **(B)** 20, **(C)** 30, and **(D)** 40 per year. Covariates used in the regression were hypertension, sex and population count, proportion of population above 60 and SES decile according to the Index of Relative Socioeconomic Advantage and Disadvantaged (IRSAD).

There is a potential problem with identifying hot spots in rural regions where the TIA count is low. A related issue is the strong dependency of standardized TIA ratio on the TIA count and population within the LGA (27). This issue arises because the boundary for the areal unit or LGA is arbitrary and shifting of the boundary may change the results. We conducted a sensitivity analysis by merging adjacent LGAs from the smallest count of TIA to reach threshold numbers of TIAs of 10, 20, 30, and 40 for each year analyzed (Figure 4) and compared the effort of this merge to the hot spots identified in rural regions.

## Results

In previous analysis, we had provided the total TIA rate and recurrence and we refer to that paper for further details (6). The characteristics of the cohort are described in Table 1. In brief, the proportion of patients with TIA above the age of 75 over the years varied from 0.57 to 0.60 and TIA above the age of 60 varied from 0.85 to 0.89. The proportion of males over the years varied from 0.47 to 0.51. The yearly trend in the number of TIA and corresponding 95% confidence interval can be seen in Figure 1. Data for the 79 LGA is provided in the Supplementary material. This shows an increase in TIA rates over the decade, which is proportional to the population increase. After adjusting to population, our previous paper showed a decline in TIA incidence for this data.

**Table 1:** Basic characteristics table over the years.

**Variables**	**2001**	**2002**	**2003**	**2004**	**2005**	**2006**	**2007**	**2008**	**2009**	**2010**
Standardized TIA ratio	1.16 ± 0.1	1.1 ± 0.14	1.16 ± 0.12	1.08 ± 0.12	1.11 ± 0.11	1.08 ± 0.14	1.17 ± 0.09	1.15 ± 0.12	1.17 ± 0.09	1.09 ± 0.09
Proportion of TIAs above 75	0.59 ± 0.14	0.62 ± 0.18	0.6 ± 0.19	0.58 ± 0.17	0.6 ± 0.19	0.58 ± 0.18	0.57 ± 0.18	0.59 ± 0.19	0.56 ± 0.16	0.55 ± 0.14
Proportion of TIAs above 60	0.89 ± 0.12	0.88 ± 0.12	0.88 ± 0.12	0.86 ± 0.09	0.85 ± 0.15	0.88 ± 0.11	0.85 ± 0.15	0.84 ± 0.1	0.84 ± 0.12	0.84 ± 0.12
Proportion of TIAs with hypertension,	0.2 ± 0.1	0.22 ± 0.14	0.24 ± 0.12	0.26 ± 0.12	0.26 ± 0.11	0.3 ± 0.14	0.28 ± 0.09	0.24 ± 0.12	0.21 ± 0.09	0.18 ± 0.09
Proportion of TIAs male	0.47 ± 0.13	0.5 ± 0.11	0.48 ± 0.11	0.5 ± 0.12	0.48 ± 0.12	0.49 ± 0.12	0.51 ± 0.11	0.49 ± 0.11	0.5 ± 0.1	0.49 ± 0.12
SES decile, median (IQR)	7 ± 4	7 ± 4	7 ± 4	7 ± 4	7 ± 4	7 ± 4	7 ± 4	7 ± 4	7 ± 4	7 ± 4

TIA, Transient Ischaemic Attack; SES, Socioeconomic Status; Continuous variables were expressed using mean and standard deviation (SD) if the variable was normally distributed and using median and interquartile range (IQR) when the variable was not normally distributed. Hypothesis testing was used to compared values between the years 2001 and 2010 to describe any temporal change. Wilcoxon rank sum test was used to compare non-normally distributed variables and Student's t-test was used to compare normally distributed variable.

### Standardized TIA ratios

Here, the standardized TIA ratio for each LGA (*n* = 79) for the years 2001 to 2010 is provided in the Supplementary Table 1. Actual TIA cases compared to expected TIA cases were higher in areas of South-West, North-East and Far East Rural regions of Victoria, as well as the outskirt suburban areas of Melbourne (Figure 2). This was in comparison to the inner suburban areas of Melbourne, where the number of actual cases were similar or lower to the expected number of TIA cases. The choropleth maps of standardized TIA ratios in LGAs across the entire state showed significant hotspot in North-West rural region, with actual cases being ~5–11 times higher than the expected cases (Figure 3). This hotspot was present throughout 2001–2010 but was most pronounced between 2002 and 2005. Within Greater Melbourne, standardized TIA ratios showed a comparably higher rate in the outskirt suburban areas, especially in the South-East regions, with actual cases being ~2 times higher than the expected cases (Figure 3). This was also constant throughout the years. A sensitivity analysis was performed to explore the impact of increasing the count of TIA in LGA on location of hot spots (Figure 4). This analysis showed that a threshold of 30 TIAs per year still resulted in a hotspot in the North-West region, with the actual cases being 5–10 times higher than the expected cases. For a threshold of 40 TIAs per year, the North-West hotspot was diluted to being 1–2 times higher actual cases than expected cases.

### Spatial and spatiotemporal regression

When adding in the spatial component (ICAR, Besag-York-Mollie, Spatial lag, Leroux) to the baseline model, this increased the Marginal Likelihood, and decreased the WAIC and DIC suggesting a spatial significance (Table 2). The optimal models with the lowest WAIC and DIC and which best explained the data were the Leoux and spatial lag models. The posterior probabilities of standardized TIA ratios for the entire State and Greater Melbourne measured using the best performing spatial models were plotted in Figures 2, 3 respectively.

**Table 2:** Spatiotemporal analysis of standard TIA ratios in local government areas in Victoria.

	**Victoria**			**Greater Melbourne**			
**Model**	**Marginal likelihood**	**WAIC**	**DIC**	**Marginal likelihood**	**WAIC**	**DIC**	**Best model**
Spatial regression							
Fixed (baseline)	−6,628	13,261	13,159	−4,325	8,689	8,557	
Spatial 1-Intrinsic conditional autoregression	−3,425	6,667	6,463	−1,617	3,147	3,001	
Spatial 2-Besag-York-Mollie	−5,025	6,665	6,462	−2,243	3,147	3,001	
Spatial 3-Leroux and others	−4,292	6,665	6,461	−1,950	3,146	3,001	*
Spatial 4-Spatial lag model	−3,435	6,665	6,462	−1,619	3,146	3,001	*
Spatiotemporal regression							
Spatial 1 + Temporal 1	−3,461	6,707	6,483	−1,643	3,166	3,001	
Spatial 1 + Temporal 2	−3,438	6,683	6,471	−1,624	3,151	2,994	
Spatial 2 + Temporal 1	−5,061	6,705	6,482	−2,269	3,165	3,001	
Spatial 2 + Temporal 2	−5,038	6,681	6,470	−2,249	3,150	2,994	
Spatial 3 + Temporal 1	−4,329	6,705	6,481	−1,977	3,165	3,001	
Spatial 3 + Temporal 2	−4,303	6,682	6,471	−1,958	3,150	2,994	
Spatial 4 + Temporal 1	−3,470	6,704	6,481	−1,646	3,164	3,001	
Spatial 4 + Temporal 2	−3,441	6,683	6,471	−1,626	3,150	2,994	

Spatiotemporal analysis of standard TIA ratios in local government areas in Victoria. Stepwise inclusion of spatial and temporal components in our Poisson regression model. Fixed, original Poisson regression model adjusted to hypertension, sex and LGA population count, proportion above age of 60, and socioeconomic status; Spatial component were either intrinsic conditional autoregression (ICAR), Besag-York-Mollie (BYM), Leroux et al. (Leroux), or spatial lag model (SLM). Temporal components were either random walk of order 1 (Temporal rw1) or autoregression of order 1 (ar1). Models were compared using marginal likelihood, Wakanabe-Akaike information criteria (WAIC) and deviance information criterion (DIC). The optimal models are represented by ^*^ sign and are chosen as they have the lowest DIC and WAIC.

There was no improvement to the spatial model after adding the temporal component, suggesting a lack of temporal trend at regional level.

## Discussion

In this disease mapping study, we examined TIA as spatial and spatiotemporal problems. Importantly, we showed that there was a strong spatial component to TIA incidence with hot spots observed around the North-West rural region and South East of Greater Melbourne. Although we had observed a decreasing trend in TIA incident at a State level (6), we did not observe any trend over a 10-year period at a regional level. Our findings provide a strategy to detect regional variation in disease as well as change in trend over time. This strategy can be used to monitor and implement preventative strategies at macro (State or Country) and micro (regional) level.

In this analysis, we had used a standard approach to spatial and spatiotemporal regression. Other investigators chosen apriori models such conditional autoregressive model (using neighbourbood weight) or Besag-York-Mollie model (neighborhood weight with unstructured random effect) (28). We have chosen to evaluate different spatial models sequentially as some models might be better suited given the location and influence of neighboring LGAs. This was the reason behind evaluating models starting from conditional autoregressive model to spatial lag model. In our analysis, the spatial lag and Leroux models best describe the Victorian data.

Our spatial model showed hot spots in North-West rural region of Victoria, and the South-Eastern Greater Melbourne. Our sensitivity analyses showed that the North-West cluster was significant even after combining multiple LGA with a threshold of 40 TIA per year per region. This unique structural pattern may be due to the remote areas of these LGA, with a lack of hospitals close to these areas. This aspect, further combined with the older population within these LGA, may explain the higher TIA rate. Our findings raised the Specter of the Stroke Belt in South-Eastern USA (14). A large proportion (31%) of people in Stroke Belt region live in rural areas. In depth studies on the Stroke Belt revealed lifestyle factors including dietary habits (high consumption of fried food, meat, processed meat, egg based dishes, high sugar content in food and drinks), lower socioeconomic status, smoking as contributors to this finding (14). By contrast, physical activity was similar across the regions and not a contributor to the high risk of stroke in South Eastern USA (14). In Canada, isolated hot spot exists in rural Canada rather than stroke belt phenomenon (9). A consistent theme is the association of stroke among regions with lower socioeconomic group even though the distribution of socioeconomic groups has remained stable (9). In Denmark, stroke and heart disease rates are higher on the Eastern part of Zealand than Eastern part of Jutland (28).

Our models did not show any significant change over time, with the hot spots remaining similar over the 10 years. This further adds to our discussion point that whilst the overall stroke recurrence after TIA decreased over 2001–2011 (6), the distribution in each location remained the same over the years. Our findings may raise the possibility primary and secondary prevention may not be optimal or possible in some regions in rural areas and Greater Melbourne. A study in Canada has highlighted that risk factors were more common and less likely to be well managed in rural than in urban residents without previous stroke episodes (29). Studies evaluating changes at regional over 10 years are not common. One such study described stability of observed pattern of high stroke risk in South Eastern USA (30). Importantly, this study compare the estimates in each county for 1995–1996 with the estimates for 2005–2006 (30). In Figure 2, we have illustrated the issue of comparing moment to moment changes in TIA rates for selected regions in rural and Greater Melbourne. It would be valuable to apply a similar method to the North American data to describe the temporal trend in each county (30).

### Limitations

The advantage of spatial model with aggregate data **(ecological model)** is that it can be performed using administrative data such as what we have done here. While our analyses were conducted retrospectively, the routine administrative data contributing to our analyses were collected prospectively. There are some limitations to this type of approach as the data used are not at the individual level but is based on aggregate count of TIA for each LGA.

Linear mixed model is one approach to the use multilevel modeling and which can include personal risk factors. We did consider the use of mixed model analysis which allow grouping of patients for each LGA. However, this approach does not recognize the neighborhood of adjacent LGA and require additional terms to describe this hierarchical structure.

As such the findings based on aggregate data and not individual data can be susceptible to the ecological inference fallacy and require further evaluation. While we have individual patient data, we cannot analyze at a person level in terms of covariate such as age, sex, hypertension and socioeconomic group. The models used aggregate count of hypertension, age above 60, males and socioeconomic group. This limitation require caution in the extrapolation of the findings to the individual. However, this method can be used to monitor regional hot spots and trend on a yearly level for the State of Victoria or at national level.

Another issue with the use of large areal unit such as LGA is that geocoding of addresses for the individuals are not available in Victoria, likely due to privacy concerns. In the absence of near approximation of addresses, we could not convert location data into smaller areal units than LGA. For examples, LGA can encompass several suburbs, with different postal addresses. Such conversion of large areal unit to smaller areal unit is possible in some European countries and in North America (28, 31). Our finding of hot spots for TIA in Victoria is exploratory in nature and needs to be replicated in independent dataset. Further evaluation of local data is required to explain why hot spot occurs in this region and not in other rural regions. This is an important caveat when using spatial model as they should be considered as exploratory rather than confirmatory. Whilst we adjusted our models to certain characteristics, there may still be unknown or unmeasured underlying confounding variables that may further explain the distribution. Our analyses could be, in the future, strategically supplemented by targeted cohort studies of risk factor management in regional hot spots that are identified. Examples of such works can be seen in papers describing the evaluation of the Stroke Belt in USA (14, 30, 32). In our State, data on patient location is coded as LGA and not smaller areal units such as suburb (with postal addresses). This issue limits our ability to perform a more detailed analysis such as the ones by North American investigators (32). Suburbs are smaller in size than LGA but there is no direct conversion between the two forms.

Finally, as highlighted previously, the diagnostic coding of TIA may have some misclassifications, including patients presenting with TIA mimics (33). This will always be the case when using administrative dataset where we do not have a chance to go back and review the diagnosis. However, given the same coding structure was utilized for the analysis, it is assumed that the error would be distributed evenly among the LGA.

## Conclusion

Our findings show hot spots in regional Victoria and within Melbourne but no yearly trends. We propose that this exploratory method may be of use to detect hot spots follow by detailed evaluation to identify opportunities to improve primary prevention of stroke, and stroke care.

## Data availability statement

The raw data supporting the conclusions of this article will be made available by the authors, without undue reservation.

## Ethics statement

The studies involving human participants were reviewed and approved by Monash University Human Research Ethics Committee. Written informed consent for participation was not required for this study in accordance with the national legislation and the institutional requirements.

## Author contributions

AK, SH, and TP performed statistical analyses. VKS and VS obtained the data. AK, SH, TP, RB, VKS, VS, and HM participate in writing of this article. All authors contributed to the article and approved the submitted version.

## Funding

The project received funding from a Pfizer Australia Cardiovascular Lipid Research Grant, 2010. The funder was not involved in the study design, collection, analysis, interpretation of data, the writing of this article or the decision to submit it for publication.

## Conflict of interest

Author TP received honoraria payments for lectures from Bayer, Pfizer, BMS, Genzyme, and Boehringer Ingelheim. The remaining authors declare that the research was conducted in the absence of any commercial or financial relationships that could be construed as a potential conflict of interest.

## Publisher's note

All claims expressed in this article are solely those of the authors and do not necessarily represent those of their affiliated organizations, or those of the publisher, the editors and the reviewers. Any product that may be evaluated in this article, or claim that may be made by its manufacturer, is not guaranteed or endorsed by the publisher.
