# Supplementary material for: Spatiotemporal analysis of regional TIA trends
Source: Front Neurol. 2022 Aug 22;13:983512. doi: 10.3389/fneur.2022.983512 (PMC9441554; doi:10.3389/fneur.2022.983512)
Supplement: Supplementary file 1 [file Table_1.pdf]

Supplementary Material

Table 1: Standardized TIA ratios over the years

Victoria Local Government Area (LGA)	LGA code	LG_PLY_PID	2001	2002	2003	2004	2005	2006	2007	2008	2009	2010
ALPINE SHIRE	20110	451.00	1.43	0.34	2.26	2.12	2.03	1.21	0.72	2.24	1.40	0.50
ARARAT RURAL CITY	20260	479.00	1.21	1.12	1.43	1.48	1.55	1.95	2.58	1.56	2.18	1.74
BALLARAT CITY	20570	447.00	1.29	1.35	1.10	1.17	1.26	1.23	0.92	1.18	1.40	0.94
BANYULE CITY	20660	392.00	1.21	0.84	0.93	1.05	1.45	1.37	1.16	1.13	1.01	0.81
BASS COAST SHIRE	20740	356.00	2.83	1.78	1.48	2.10	1.54	1.82	2.14	1.89	2.11	2.26
BAW BAW SHIRE	20830	515.00	0.90	0.40	0.66	0.59	0.84	0.82	1.30	1.26	0.84	0.97
BAYSIDE CITY	20910	307.00	1.49	1.67	1.70	1.66	1.40	1.06	1.50	1.49	1.46	1.06
BENALLA RURAL CITY	21010	427.00	0.00	0.00	0.00	0.00	1.18	1.72	2.02	1.16	1.90	1.20
BOROONDARA CITY	21110	455.00	0.78	0.97	0.90	0.79	0.77	0.73	0.76	0.68	0.81	0.66
BRIMBANK CITY	21180	497.00	0.80	0.77	0.62	0.77	0.90	0.86	0.87	0.86	0.79	0.81
BULOKE SHIRE	21270	435.00	3.30	1.60	1.90	0.88	1.90	2.99	1.71	5.44	1.60	2.29
CAMPASPE SHIRE	21370	440.00	1.59	1.94	1.64	2.29	2.21	1.24	1.55	1.39	2.17	1.76
CARDINIA SHIRE	21450	516.00	0.48	0.78	0.51	0.47	0.48	0.92	0.97	0.76	0.61	0.63
CASEY CITY	21610	517.00	0.46	0.43	0.52	0.61	0.48	0.64	0.58	0.72	0.44	0.74
CENTRAL GOLDFIELDS SHIRE	21670	423.00	1.52	2.01	2.23	2.71	1.66	1.43	1.41	1.41	2.21	1.44
COLAC OTWAY SHIRE	21750	488.00	0.88	1.67	0.94	2.62	2.29	1.66	0.85	1.31	1.26	0.73
CORANGAMITE SHIRE	21830	475.00	1.54	0.92	2.29	1.09	1.14	1.59	1.32	2.41	1.95	2.20
DAREBIN CITY	21890	502.00	1.17	1.29	1.22	1.08	1.01	1.10	0.98	0.98	1.00	0.65
EAST GIPPSLAND SHIRE	22110	466.00	2.02	2.24	1.75	2.10	1.55	1.94	1.76	1.24	1.66	1.43
FRANKSTON CITY	22170	481.00	1.89	1.55	1.63	1.61	1.50	1.33	1.57	1.56	1.87	1.66
GANNAWARRA SHIRE	22250	506.00	1.53	1.46	1.54	1.61	1.42	3.38	2.62	1.31	1.84	1.13
GLEN EIRA CITY	22310	281.00	1.30	1.01	1.26	1.15	1.08	1.04	0.92	0.92	0.85	0.97
GLENELG SHIRE	22410	509.00	3.07	2.65	1.94	1.31	2.95	1.26	1.69	1.92	2.08	1.98
GOLDEN PLAINS SHIRE	22490	484.00	0.66	0.66	0.58	0.49	0.54	0.80	0.86	0.37	0.69	0.41
GREATER BENDIGO CITY	22620	510.00	0.91	0.94	0.97	0.75	1.24	1.08	1.18	1.14	1.40	1.40
GREATER DANDENONG CITY	22670	283.00	0.72	0.82	0.78	0.75	0.60	0.99	0.81	1.08	0.96	0.94
GREATER GEELONG CITY	22750	471.00	0.75	0.72	0.73	0.94	0.87	1.21	0.97	1.18	1.39	1.05

GREATER SHEPPARTON CITY	22830	513.00	0.78	1.36	0.90	1.25	0.96	1.24	1.17	1.07	1.45	1.58
HEPBURN SHIRE	22910	500.00	1.28	1.52	1.38	1.98	0.85	0.96	2.31	1.47	1.28	1.77
HINDMARSH SHIRE	22980	344.00	5.17	1.56	5.41	3.95	4.06	1.94	2.17	2.66	4.87	1.78
HOBSONS BAY CITY	23110	490.00	0.93	0.86	0.68	1.12	1.07	0.96	0.85	1.03	0.71	0.97
HORSHAM RURAL CITY	23190	469.00	1.15	1.80	0.82	1.77	1.35	2.13	1.40	1.60	1.36	1.49
HUME CITY	23270	287.00	0.55	0.60	0.54	0.63	0.62	0.66	0.75	0.72	0.67	0.65
INDIGO SHIRE	23350	492.00	0.58	0.49	0.41	1.15	0.89	0.89	1.17	0.74	1.42	1.09
KINGSTON CITY	23430	480.00	1.34	1.36	1.30	1.28	1.09	1.20	1.23	1.19	1.22	1.22
KNOX CITY	23670	478.00	0.14	0.10	0.14	0.12	0.08	0.11	0.13	0.11	0.18	1.16
LATROBE CITY	23810	429.00	1.13	1.07	0.82	0.64	0.74	0.69	1.05	1.07	1.58	0.91
LODDON SHIRE	23940	508.00	1.65	0.68	2.35	0.95	2.03	0.75	2.24	3.70	2.59	4.55
MACEDON RANGES SHIRE	24130	432.00	0.83	0.80	1.18	0.88	0.65	0.53	0.88	0.95	1.08	0.79
MANNINGHAM CITY	24210	458.00	0.95	0.92	0.98	0.79	1.12	1.06	0.91	0.97	0.94	0.92
MANSFIELD SHIRE	24250	464.00	0.00	0.00	0.00	0.00	1.45	0.81	1.38	1.27	1.99	0.77
MARIBYRNONG CITY	24330	292.00	1.17	1.14	0.72	0.93	1.15	0.86	0.85	0.57	0.73	0.66
MAROONDAH CITY	24410	504.00	0.96	1.22	1.14	0.97	1.03	1.46	1.27	1.45	1.21	1.02
MELBOURNE CITY	24600	419.00	0.45	0.40	0.11	0.28	0.43	0.30	0.40	0.34	0.36	0.34
MELTON CITY	24650	496.00	0.53	0.52	0.49	0.47	0.60	0.50	0.34	0.64	0.53	0.43
MILDURA RURAL CITY	24780	430.00	1.29	0.90	0.82	0.72	1.10	0.62	0.90	0.78	0.69	1.20
MITCHELL SHIRE	24850	405.00	0.97	1.27	0.82	1.55	1.12	1.30	0.98	0.70	0.66	0.98
MOIRA SHIRE	24900	491.00	1.91	1.58	1.52	1.68	1.37	0.70	1.96	1.43	1.59	1.93
MONASH CITY	24970	295.00	0.91	1.03	1.19	0.98	1.00	1.03	1.12	0.97	1.03	0.99
MOONEE VALLEY CITY	25060	518.00	1.18	1.10	1.16	1.20	0.92	1.14	1.14	1.11	1.17	0.93
MOORABOOL SHIRE	25150	457.00	0.73	1.15	1.31	0.73	0.40	0.97	0.67	0.42	0.90	1.03
MORELAND CITY	25250	501.00	1.23	1.20	1.13	1.60	1.20	1.26	1.37	1.23	1.03	1.06
MORNINGTON PENINSULA SHIRE	25340	498.00	2.33	2.19	2.65	2.42	2.60	2.41	2.11	2.22	2.32	1.93
MOUNT ALEXANDER SHIRE	25430	499.00	1.33	1.52	0.87	1.26	2.66	1.11	0.84	1.19	1.48	2.13
MOYNE SHIRE	25490	340.00	1.71	1.84	1.92	2.18	1.80	1.13	1.03	1.52	0.87	1.70
MURRINDINDI SHIRE	25620	378.00	2.11	2.58	1.23	1.62	1.54	1.96	1.39	1.28	0.92	1.93
NILLUMBIK SHIRE	25710	512.00	0.42	0.28	0.35	0.56	0.61	0.72	0.45	0.39	0.42	0.39

NORTHERN GRAMPIANS SHIRE	25810	476.00	1.31	1.23	2.49	0.99	1.93	2.45	2.43	2.52	2.93	3.38
PORT PHILLIP CITY	25900	505.00	0.86	0.49	0.59	0.54	0.40	0.65	0.71	0.62	0.55	0.38
PYRENEES SHIRE	25990	450.00	1.28	2.41	1.60	2.12	1.34	1.34	0.88	1.69	1.15	0.90
QUEENSCLIFFE BOROUGH	26080	413.00	0.43	0.90	1.42	2.48	2.38	1.44	2.38	4.17	2.52	2.97
SOUTH GIPPSLAND SHIRE	26170	426.00	1.68	1.45	2.48	1.49	1.25	1.19	1.56	1.33	0.86	1.12
SOUTHERN GRAMPIANS SHIRE	26260	398.00	1.41	1.96	2.22	1.74	2.45	2.02	1.83	0.86	2.02	2.27
STONNINGTON CITY	26350	425.00	1.13	0.84	0.90	0.89	1.00	0.68	0.72	0.90	0.60	0.57
STRATHBOGIE SHIRE	26430	514.00	2.49	1.81	2.50	2.28	4.54	2.65	2.77	1.18	1.61	3.16
SURF COAST SHIRE	26490	489.00	0.89	0.75	0.56	0.94	0.95	0.80	0.32	0.53	0.80	0.64
SWAN HILL RURAL CITY	26610	383.00	3.40	2.86	1.35	2.21	1.55	2.34	1.47	2.45	1.47	2.24
TOWONG SHIRE	26670	410.00	1.58	1.16	3.41	2.27	2.42	0.97	0.48	1.33	2.30	2.27
WANGARATTA RURAL CITY	26700	433.00	1.23	1.85	1.76	1.35	0.89	1.44	2.24	1.25	1.25	1.45
WARRNAMBOOL CITY	26730	304.00	1.44	1.79	1.35	0.97	1.45	1.68	2.06	1.87	2.36	1.59
WELLINGTON SHIRE	26810	428.00	0.72	1.34	1.51	1.34	1.32	1.02	1.69	2.38	1.30	1.38
WEST WIMMERA SHIRE	26890	507.00	5.82	4.23	5.09	2.34	1.94	3.60	2.62	3.25	3.84	0.69
WHITEHORSE CITY	26980	460.00	0.98	1.16	1.40	1.19	1.21	1.38	1.41	1.15	1.16	1.38
WHITTLESEA CITY	27070	511.00	0.11	0.08	0.11	0.09	0.07	0.16	0.15	0.12	0.12	0.52
WODONGA CITY	27170	493.00	0.88	0.84	0.82	0.92	1.06	0.93	0.91	1.27	0.80	1.37
WYNDHAM CITY	27260	470.00	0.03	0.67	0.59	0.40	0.49	0.43	0.47	0.39	0.51	0.47
YARRA CITY	27350	306.00	0.53	0.21	0.41	0.52	0.47	0.47	0.53	0.55	0.28	0.52
YARRA RANGES SHIRE	27450	503.00	0.14	0.19	0.22	0.13	0.16	0.14	0.20	0.24	0.22	0.79
YARRIAMBIACK SHIRE	27630	474.00	5.30	11.36	9.57	8.09	9.59	3.12	4.70	2.16	5.00	4.56
